# The Antioxidant and Anti-inflammatory Effects of Phenolic Compounds Isolated from the Root of *Rhodiola sachalinensis* A. BOR

**DOI:** 10.3390/molecules171011484

**Published:** 2012-09-27

**Authors:** Kang In Choe, Joo Hee Kwon, Kwan Hee Park, Myeong Hwan Oh, Manh Heun Kim, Han Hyuk Kim, Su Hyun Cho, Eun Kyung Chung, Sung Yi Ha, Min Won Lee

**Affiliations:** College of Pharmacy, Chung-Ang University, Seoul 156-756, Korea

**Keywords:** *Rhodiola sachalinensis*, antioxidants, radical scavengers, Nitric oxide, HPLC

## Abstract

Isolation of compounds from the root of *Rhodiola sachalinensis *(RRS) yielded tyrosol (**1**), salidroside (**2**), multiflorin B (**3**), kaempferol-3,4′-di-*O*-β-D-glucopyranoside (**4**), afzelin (**5**), kaempferol (**6**), rhodionin (**7**), and rhodiosin (**8**). Quantification of these compounds was performed by high-performance liquid chromatography (HPLC). To investigate the antioxidant and anti-inflammatory effects of the compounds, DPPH radical scavenging, NBT superoxide scavenging and nitric oxide production inhibitory activities were examined in LPS-stimulated Raw 264.7 cells. We suggest that the major active components of RRS are herbacetin glycosides, exhibiting antioxidant activity, and kaempferol, exhibiting anti-inflammatory activity.

## 1. Intoduction

*Rhodiola sachalinensis* A. BOR belongs to the family Crassulaceae, and the root of the plant (RRS) is popular in traditional medical systems in Siberia and Asia. RRS has been used to treat flu-like symptoms and fatigue and is known to prevent high altitude sickness [1,2,3]. Additionally, it has been reported that extracts of RRS and compounds isolated from RRS possess antioxidant [4], anti-inflammatory [5], hepatoprotective [6], sedative, hypnotic [7] and anti-fatigue effects [8]. Various phenolic compounds are found in RRS, such as phenyl propanoids [9], flavonoids and gallic acid derivatives [10]. In this report, we describe the isolation, analysis, and quantification of the phenolic compounds in RRS, followed by an evaluation of the antioxidant and anti-inflammatory activities of these compounds.

## 2. Results and Discussion

### 2.1. Isolation and Identification

**Figure 1:**
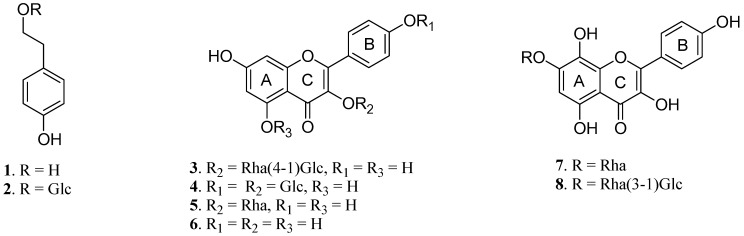
Chemical structures of compounds **1**-**8** isolated from RRS.

RRS were extracted with 80% acetone, and the extract was subjected to a combination of MCI-gel CHP 20P, ODS-B gel and Sephadex LH-20 chromatographies for fractionation. Repeated column chromatography was performed for each fraction to yield tyrosol (**1**) [11], salidroside (**2**) [12], multiflorin B (**3**) [13], kaempferol-3,4′-di-*O*-β-D-glucopyranoside (**4**) [14], afzelin (**5**) [15], kaempferol (**6**) [10], rhodionin (**7**) and rhodiosin (**8**) [16], respectively. The structures of the isolated compounds (Figure 1) were identified by comparing their NMR spectral data with values reported in the literature.

### 2.2. Quantitative Analysis by HPLC

**Figure 2:**
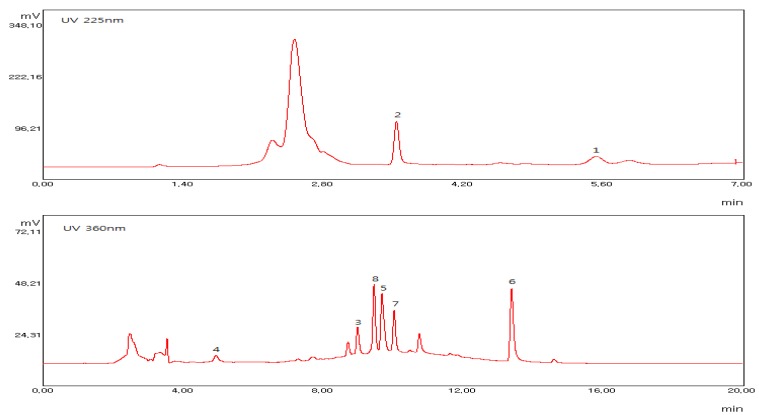
HPLC chromatograms of RRS extracts.

The HPLC resolution of **1**–**8** was optimized for a mixture of standard compounds and for RRS extract. Due to the differing selective absorption wavelengths of the compounds, **1** and **2** were analyzed at a wavelength of 225 nm [17] and **3**–**8** were analyzed at a wavelength of 360 nm (Figure 2).

Calibration functions of standards of each of **1**–**8** were calculated with peak area (y) and concentration (x) in μg mL^−1^ and were y = 11.276x + 25.91 (R^2^ = 0.9948), y = 41.035x + 47.837 (R^2^ = 0.9996), y = 27.667x + 39.288 (R^2^ = 0.9963), y = 18.797x + 27.252 (R^2^ = 0.9930), y = 26.88x + 39.39 (R^2^ = 0.9984), y = 12.7x + 4.0217 (R^2^ = 0.9999), y = 12.72x + 8.6013 (R^2^ = 1.0000) and y = 11.122x + 14.22 (R^2^ = 0.9990), respectively. The most abundant compounds detected in RRS were **2** (22.54 mg per 1g of dry extract), **6** (8.94 mg per 1 g of dry extract), **7 **(3.98 mg per 1 g of dry extract) and **8** (7.13 mg per 1g of dry extract) (Table 1).

**Table 1:** Quantitative data for extracts of RRS.

Compound	Concentration (mg g^−1^ dry extract)
**1**	2.39
**2**	22.54
**3**	1.00
**4**	0.40
**5**	2.82
**6**	8.94
**7**	3.98
**8**	7.13

### 2.3. Antioxidant Activity

In this study, 1,1-diphenyl-2-picryl-hydrazyl (DPPH) radical scavenging activity and nitrotetrazolium blue chloride (NBT) superoxide scavenging activity were measured to assess the antioxidant activity of the components from RRS. DPPH has the ability to easily accept hydrogen atoms because it contains an unstable element, the hydrazyl nitrogen, therefore, antioxidant activity can be measured because DPPH loses its violet color when it receives hydrogens from antioxidants [18]. Additionally, NBT has the ability to easily receive superoxide because it contains unstable anions. Therefore, antioxidant activity may be measured when NBT loses its yellow color upon reaction with abundant superoxide [19]. 

Among the compounds from RRS, **7** and **8** exhibited the most potent DPPH free radical scavenging activities, with IC_50_ values of 19.49 ± 0.21 and 27.77 ± 0.61 μM, respectively, compared to the positive control, L-ascorbic acid (IC_50_ = 32.89 ± 0.70 μM). The other compounds did not exhibit activities in this assay up to 100 μM (Table 2).

**Table 2:** DPPH radical scavenging activities, NBT/superoxide scavenging activities and inhibitory effects against NO production of each compound extracted from RRS.

Compound	DPPH radical scavenging activity IC_50_ (μM)	NBT/superoxide scavenging activity IC_50_ (μM)	Inhibitory activity on NO production IC_50_ (μM)
**1**	>100	>100	>100
**2**	>100	>100	>100
**3**	>100	>100	>100
**4**	>100	>100	>100
**5**	>100	>100	>100
**6**	>100	>100	21.34 ± 2.52 ^b^
**7**	19.49 ± 0.21 ^a^	13.19 ± 3.27 ^b^	>100
**8**	27.77 ± 0.61 ^b^	9.92 ± 0.77 ^a^^,b^	>100
L-ascorbic acid	32.89 ± 0.70 ^c^	-	-
Allopurinol	-	7.03 ± 0.42 ^a^	-
L-NMMA	-	-	8.57 ± 2.76 ^a^

Values represent means ± S.D. of three determinations. Values bearing different superscripts in the same column are significantly different (*p *< 0.05). -, Not determined.

The NBT superoxide scavenging activity of compounds from RRS was similar to the DPPH free radical scavenging activity, as **7** and **8** showed moderate NBT superoxide scavenging activities with IC_50 _values of 13.19 ± 3.27 and 9.92 ± 0.77 μM, respectively, compared to the positive control, L-allopurinol (IC_50_ = 7.03 ± 0.42 μM). Again, the other compounds did not show activities in this assay up to 100 μM level (Table 2). These two assessments show that the herbacetin glycosides (**7** and **8**), which possess an 8-OH on an A-ring, have better antioxidant effects than kaempferol glycosides (**3**, **4** and **5**). This result also suggests that the 8-OH group in the herbacetin glycosides might be crucial to the antioxidant activity of the RSS-derived compounds.

### 2.4. Inhibitory Activity on NO Production

The inhibitory activity of each compound against nitric oxide (NO) production was evaluated in the RAW 264.7 cell line treated with lipopolysaccharide (LPS). LPS treatment induces over-expression of inducible nitric oxide synthase (iNOS) in order to create inflammation [20]. Among the compounds isolated from RRS, **6** (IC_50_ = 21.34 ± 2.52 μM) moderately reduced NO production compared to the positive control, NG-monomethyl-L-arginine (L-NMMA) (IC_50_ = 8.57 ± 2.76 μM) (Table 2). Compound **6** is known to inhibit the expressions of iNOS and cyclooxygenase-2 (COX-2) [21]. This result differed from the results of the antioxidant activity, perhaps because compounds **7** and **8** possess glycoside moieties, which have difficulty entering the intracellular environment.

### 2.5. Cell Viability

Cell viability was measured using the 3-(4,5-dimethylthiazol-2-yl)-2,5-diphenyltetrazolium-bromide (MTT) assay, which is based on the mitochondria-dependent reduction of MTT to formazan [22]. None of the compounds exhibited cytotoxicity in the RAW 246.7 cell line at the experimental doses (12.5-100 μM), demonstrating that the inhibitory effects on NO production of these compounds were unrelated to their cytotoxicity (data not shown).

## 3. Experimental

### 3.1. General Procedures

The stationary phases for the column chromatographic isolation were Sephadex LH-20 (25–100 μm; GE Healthcare Bio-Science AB, Uppsala, Sweden), MCI-gel CHP 20P (75–150 μm; Mitsubishi Chemical, Tokyo, Japan) and ODS-B gel (40–60 μm; Daiso, Osaka, Japan). ODS-B gel was used as the stationary phase on the medium pressure liquid chromatography (MPLC) system. The sample injector used was a Waters 650E (Waters, Milford, MA, USA), the detector used was the 110 UV/VIS detector (Gilson, Middleton, WI, USA) and the pump used was the TBP5002 (Tauto Biotech, Shanghai, China). Thin layer chromatography (TLC) was carried out using pre-coated silica gel 60 F_254_ plates (Merck, Darmstadt, Germany) eluted with chloroform, methanol and water (70:30:4 volume ratio). The spots were detected under UV radiation (254 nm) and by spraying with FeCl_3_ and 10% H_2_SO_4_ or anisaldehyde-H_2_SO_4_ followed by heating. The components from the roots of *Rhodiola sachalinensis *A. Bor were identified by ^1^H-NMR (300 or 600 MHz) recorded using a Gemini 2000 and VNS (Varian, CA, USA) at the center for research facilities at Chung-Ang University. The chromatographic system for quantitative analysis consisted of a Waters 600 system controller, Waters 600 pump, Waters 112 UV/VIS detector (Waters, Milford, MA, USA) and Autochro-Win 3.0 plus data system (Young-lin Co., Anyang, Korea). All other chemicals and solvents were of analytical grade.

### 3.2. Plant Material

The roots of *Rhodiola sachalinensis *were purchased from Kyung-dong Market, Seoul, Korea in October 2009. They were identified by Professor Lee of the College of Pharmacy in Chung-Ang University. A voucher specimen (RRS200910) was deposited at the herbarium of the College of Pharmacy, Chung-Ang University.

### 3.3. Extraction and Isolation

The roots of *Rhodiola sachalinensis *(3.0 kg) were extracted several times with 80% acetone (35.0 L) at room temperature for 72 h. Concentration, that is removal of the acetone under vacuum, afforded 953.8 g of extract. After acetone evaporation, the aqueous residue was filtered. The filtrate was concentrated and 197.5 g of the filtrate were applied to a Sephadex LH-20 column (25–100 μm, 2000 g, 10 × 120 cm). The column was eluted using a H_2_O-MeOH gradient, yielding 11 fractions. Repeated column chromatography of fraction 2, using the MCI gel CHP 20P with a gradient solvent system of H_2_O/MeOH (from 70:30 to 0:100) and low-pressure liquid column chromatography yielded **1** (769 mg). Repeated column chromatography of fraction 3, using MCI gel with a gradient solvent system of H_2_O/MeOH (from 100:0 to 0:100) yielded **2** (1,870 mg). Repeated column chromatography of fraction 6, using MCI gel CHP 20P with a gradient solvent system of H_2_O/MeOH (from 60:40 to 0:100) and ODS-B gel with a gradient solvent system of H_2_O/MeOH (from 60:40 to 0:100) yielded **3 **(124 mg). Repeated column chromatography of fraction 4, using MCI gel CHP 20P with a gradient solvent system of H_2_O/MeOH (from 100:0 to 0:100) and ODS-B gel with a gradient solvent system of H_2_O/MeOH (from 100:0 to 0:100) yielded **4** (66 mg). Repeated column chromatography of fraction 7, using MCI gel CHP 20P with a gradient solvent system of H2O/MeOH (from 50:50 to 0:100) and ODS-B gel with a gradient solvent system of H2O/MeOH (from 60:40 to 0:100) yielded **5** (70 mg). Repeated column chromatography of fraction 10, using ODS-B gel with a gradient solvent system of H2O/MeOH (from 50:0 to 0:100) yielded **6 **(1,450 mg). Repeated column chromatography of fraction 9, using ODS-B gel with a gradient solvent system of H2O/MeOH (from 60:40 to 0:100) yielded **7 **(590 mg). Repeated column chromatography of fraction 8, using MCI gel CHP 20P with a gradient solvent system of H2O/MeOH (from 60:40 to 0:100) and ODS-B gel with a gradient solvent system of H2O/MeOH (from 50:50 to 0:100) yielded **8** (525 mg). All of isolated compounds were already known, so the structure of each compound could be identified by comparing only ^1^H-NMR spectral data with values reported in the literature.

*Tyrosol *(**1**). Colorless needle-like crystals; ^1^H-NMR (600 MHz, CD_3_OD-*d*_4_ + D_2_O): δ 7.03 (2H, d, *J* = 6.0 Hz, H-2,6), 6.72 (2H, d, *J* = 6.0 Hz, H-3,5) 3.69 (2H, t, *J* = 6.0 Hz, H-8, CH_2_), 2.72 (2H, t, *J *= 6.0 Hz, H-7, CH_2_).

*Salidroside* (**2**). Yellow amorphous powder; ^1^H-NMR (300 MHz, CD_3_OD-*d*_4_ + D_2_O): δ 7.06 (2H, d, *J *= 8.4 Hz, H-2,6), 6.69 (2H, d, *J *= 8.4 Hz, H-3,5), 4.29(1H, d, *J *= 7.8 Hz, glc-1), 4.02(1H, m, H-8a), 3.84 (1H, m, H-6′a), 3.69 (1H, m, H-8b), 3.66 (1H, m, H-6′b), 3.35-3.26(5H, glysoside proton), 2.83(2H, t, *J* = 7.2 Hz, H-7).

*Multiflorin B* (**3**). Yellow amorphous powder; ^1^H-NMR (300 MHz, CD_3_OD-*d*_4 _+ D_2_O): δ 7.72 (2H, d, *J* = 8.7 Hz, H-2′, 6′), 6.90 (2H, d, *J* = 8.7 Hz, H-3′, 5′), 6.43 (1H, d, *J* = 2.1 Hz, H-8), 6.15 (1H, *J* = 2.1 Hz, H-6), 5.29 (1H, m, rha-1), 4.44 (1H, d, *J* = 7.5 Hz, glc-1), 4.37(1H, m, rha-2) 3.82-3.23 (9H glycoside proton), 0.93 (3H, d, *J* = 5.4 Hz, rha-CH_3_).

*Kaempferol-3,4′-di-O-β-D-glucopyranoside *(**4**). Yellow amorphous powder; ^1^H-NMR (600 MHz, DMSO-*d*_6_ + D_2_O): δ 8.08 (2H, d, *J* = 9.0 Hz, H-2′, 6′), 7.12 (2H, d, *J* = 9.0 Hz, H-3′, 5′), 6.43 (1H, m, H-8), 6.20 (1H, m, H-6), 5.43 (1H, d, *J* = 6.0 Hz, 3′-glc-1), 4.99 (1H, d, *J* = 6.0 Hz, 4′-glc-1′), 3.69–3.07 (12H, glycoside proton).

*Afzelin *(**5**). Brown amorphous powder; ^1^H-NMR (600 MHz, DMSO-*d*_6_ + D_2_O): δ 7.71 (2H, d, *J* = 9.0 Hz, H-2′, 6′), 6.93 (2H, d, *J* = 9.0 Hz, H-3′, 5′), 6.36 (1H, d, *J* = 2.4 Hz, H-8), 6.19 (1H, d, *J* = 2.4 Hz, H-6), 5.35 (1H, d, *J* = 1.8 Hz, rha-1), 4.23(1H, dd, *J* = 1.2, 1.8 Hz, rha-2), 3.72(1H, m, rha-2), 3.34–3.30 (2H, glycoside proton), 0.92 (3H, d, *J* = 6.0 Hz, rha-CH_3_).

*Kaempferol* (**6**). Yellow amorphous powder; ^1^H-NMR (300 MHz, DMSO-*d*_6_ + D_2_O): δ 8.02 (2H, d, *J* = 8.7 Hz, H-2′, 6′), 6.92 (2H, d, *J* = 8.7 Hz, H-3′, 5′), 6.44 (1H, m, H-8), 6.19 (1H, m , H-6).

*Rhodionin* (**7**). Green amorphous powder; ^1^H-NMR (600 MHz, DMSO-*d*_6_ + D_2_O): δ 8.14 (2H, d, *J* = 9.0 Hz, H-2′, 6′), 6.95 (2H, d, *J* = 9.0 Hz, H-3′, 5′), 6.59 (1H, s, H-6), 5.48 (1H, m, rha-1), 4.95 (1H, m, rha-2), 3.82 (1H, m, rha-5), 3.33 (1H, m, rha-4), 1.26 (3H, d, *J* = 6.0 Hz, rha-CH_3_).

*Rhodiosin *(**8**). Yellow amorphous powder; ^1^H-NMR (600 MHz, DMSO-*d*_6_ + D_2_O): δ 8.11 (2H, d, *J* = 6.0 Hz, H-2′, 6′), 6.92 (2H, d, *J* = 6.0 Hz, H-3′, 5′), 6.58 (1H, s, H-6), 5.51 (1H, s, rha-1), 4.49 (1H, d, *J* = 7.45 Hz, glc-1), 4.18(1H, m, rha-2), 4.15–3.09 (9H, sugar proton), 1.10 (3H, d, *J* = 6.0 Hz, rha-CH_3_).

### 3.4. Quantitative Analysis by HPLC

Quantification of compounds from the roots of *Rhodiola sachalinensis *(RRS) was performed using high-performance liquid chromatography (HPLC). Samples were separated using a Kromasil 100-5C18 column (250 × 4.6 mm i.d., 5μm) (AkzoNobel, Bohus, Sweden), with the following gradient elution: 0 min, 20% A; 20 min, 100% A (A: acetonitrile, B: water) at a flow rate of 1 mL/min. The chromatograms were registered at 225 nm (**1**–**2**) and 360 nm (**6**–**8**) wavelengths. The injection volume was 20 μL. The data were integrated using the Autochro-Win 3.0 plus software system. Stock solutions of compounds **1**–**2** isolated from RRS was made by adding 0.5 mg of compound to a suitable solvent (methanol). Then standard solutions of various concentrations (50 μg mL^−1^, 25 μg mL^−1^, 12.5μg mL^−1^, 6.25 μg mL^−1^ and 3.25 μg mL^−1^) were made by dilution of the stock solution. The detection liquid was made by adding 1.5 mg of RRS extract to the solvent (methanol). Stock solution of compounds **6–8** isolated from the RRS was made by adding 1.0 mg of compound to the selected solvent (methanol). Then standard solutions of various concentrations (25 μg mL^−1^, 12.5 μg mL^−1^, 6.25 μg mL^−1^, 3.25 μg mL^−1^ and 1.625 μg mL^−1^) were made by dilution of the stock solution. The detection mixture was made by adding 2.0 mg of RRS extract to the selected solvent (methanol). Prior to analysis, all solutions were filtered through a 0.45 μm syringe filter and then injected. The linearity of the HPLC calibration curve was established for authentic samples of compounds. Quantitative determinations were carried out by peak area measurements at 225 nm (**1**–**2**) and 360 nm (**3**–**8**), using a calibration curve of compounds at the same wavelength.

### 3.5. Measurement of DPPH Radical Scavenging Activity

The antioxidant activity was determined on the basis of the scavenging activity of the stable DPPH free radical (Sigma, St. Louis, MO, USA) [18] Twenty μL of each sample in absolute ethanol were added to the DPPH solution (180 μL, 0.1 mM in absolute ethanol). After mixing gently and incubating for 30 min, the optical density was measured at 540 nm using an ELISA reader (TECAN, Salzburg, Austria). The free radical scavenging activity was calculated as the inhibition rate (%) = [1 − (sample optical density/control optical density)] × 100. The IC_50_ values were defined as the concentration that could scavenge 50% of the DPPH free radical. L-Ascorbic acid was used as a positive control.

### 3.6. Measurement of NBT/Superoxide Scavenging Activity

A reaction mixture with a final volume of 632 μL/Eppendorf tube was prepared with 50 mM phosphate buffer (pH 7.5) containing EDTA (0.05 mM), hypoxanthine (0.2 mM), 63 μL NBT (1 mM) (Sigma, St. Louis, MO, USA), 63 μL of aqueous or ethanolic extract (distilled water for the control), and 63 μL of xantine oxidase (1.2 U/μL, Sigma). The xanthine oxidase was added last. For each sample analysis, a blank reading was first established. The subsequent rate of NBT reduction was determined on the basis of sequential spectrophotometric determinations of absorbance at 560 nm. The solutions were prepared daily, and kept from light. The results are expressed as the percentage inhibition of the NBT reduction with respect to the reaction mixture without sample (buffer only). Superoxide anion scavenging activities were calculated by the following equation: [(1 − (sample optical density − blank optical density)/(control optical density − blank optical density)) × 100]. Activities were expressed as IC_50_ values, which were defined as the concentrations at which 50% of the NBT/superoxide anion was scavenged. Allopurinol (Sigma) was used as a positive control.

### 3.7. Cell Culture

RAW 264.7 macrophage cell lines were purchased from the Korean Cell Line Bank. The cells were grown at 37 °C in a humidified atmosphere (5% CO_2_) in DMEM (Sigma) containing 10% fetal bovine serum, 10 IU/mL penicillin G and 100 μg/mL streptomycin (Gibco BRL, Grand Island, NY, USA).

### 3.8. Measurement of Cell Viability

After culturing the RAW 264.7 macrophages (3 × 104 cells/ 200 μL medium) in 96-well plates and incubating them for 2 h, the cells were treated with the test samples. The cells were incubated for an additional 24 h, and the medium was replaced with fresh medium containing 0.5 mg/mL 3-(4,5-dimethylthiazol-2-yl)-2,5-diphenyltetrazolium bromide (MTT, Sigma). Incubation was continued for 4 h at 37 °C. The medium was then removed and the MTT-formazan produced was dissolved in dimethyl sulfoxide (DMSO). The extent of the reduction of MTT to formazan within the cells was quantified by measuring the absorbance at 540 nm using a microplate reader [20]. Cytotoxicity was calculated as cell viability (%) = sample optical density*/*blank optical density × 100.

### 3.9. Measurement of Inhibitory Activity on NO production

RAW 264.7 macrophage cells (3 × 10^4^/200 μL medium) were seeded onto 96-well plates and incubated for 2 h at 37 °C in a humidified atmosphere (5% CO_2_). The cells were then incubated in medium containing 1 μg/mL lipopolysaccharide (LPS, Sigma) and test samples. After incubating for an additional 24 h, Griess reagent (0.1% naphthylethylene-diamine and 1% sulfanilamide in 5% H_3_PO_4_ solution, Sigma) was added to supernatant obtained from cells treated with the various RRS isolates. L-NMMA was used as the positive control. The absorbance of the samples was then read at 540 nm using the microplate reader and the amount of nitrite in the samples was calculated from a sodium nitrite standard curve [23]. Inhibitory activity against NO production was calculated as inhibition rate (%) = [1 − (sample optical density − blank optical density)/(control optical density-blank optical density)] × 100, and the IC_50_ values were determined.

### 3.10. Statistical Analysis

All data are expressed as means ± S.D. Values were analyzed by one-way analysis of variance (ANOVA) followed by the Student-Newman-Keuls (S-N-K) test using the SPSS software package; the values were considered significantly different when the *p *value was less than 0.05. Values bearing different superscripts in the same column are significantly different.

## 4. Conclusions

RRS has been used as a tonic in Siberia and Asia. It has reported that salidroside, the major active component, possessed anti-fatigue effects [8,24]. We found that salidroside is indeed a major component of RRS, but was not a major active component of RRS in the context of antioxidant and anti-inflammatory activities. We suggest that the major antioxidant components of RRS are herbacetin glycosides and that the major anti-inflammatory component is kaempferol.
